# Gene therapy in ophthalmology

**DOI:** 10.4103/0974-620X.57308

**Published:** 2009

**Authors:** Satagopan Uthra, Govindasamy Kumaramanickavel

**Affiliations:** Department of Ophthalmology, Health City, Bangalore, India; 1 Narayana Nethralaya, Health City, Bangalore, India

**Keywords:** Gene therapy, ophthalmology, Leber′s congenital amaurosis

## Abstract

It has been more than a year since ophthalmologists and scientists under Dr. Robin Ali′s team at the Moorsfield Eye Hospital and the Institute of Ophthalmology, University College London, successfully treated patients with a severely blinding disease, Leber′s congenital amaurosis (LCA) using gene therapy. This success does not look to be transient, and this achievement in gene replacement therapy clinical trial for LCA has instilled hope in numerous families with patients suffering from this and similar retinal degenerative diseases, for whom restoration of lost vision has remained a distant dream so far. The encouragement that this success has given is expected to also lead to start of clinical trials for other blinding ocular diseases for which gene therapy experiments at the laboratory and animal levels have been successful. This article reviews the various studies that have led to the understanding of gene therapy outcomes in human ocular diseases and attempts to provide a brief sketch of successful clinical trials.

## Introduction

Although history of genetics dates back to more than a century, only the last few decades, encompassing the DNA and genomics era, have witnessed breakthrough research work that have revolutionized various fields including medicine. The only possible treatment that was predicted to become available for untreatable genetic disorders was gene therapy. The first ray of hope came in 1990, through successful retroviral transduction of the gene coding for adenosine deaminase in four-year-old Ashanti De Silva, who suffered from severe combined immunodeficiency (SCID). However, the trust in gene therapy was shaken by the death of 18-year-old Jesse Gelsinger in 1999 due to multiple organ failure following systemic administration of an adenoviral vector having ornithine transcarbamylase gene. Despite this, continued success in treating SCID patients and hemophilia are quite encouraging.[1]

Ocular gene therapy research has made rapid progress in the past few years. Although laboratory and animal experiments started were successful many years ago, the application in human beings took very long due to several biological and regulatory hurdles. However, the recent successful gene therapy clinical trials are promising and encouraging.[2] There are various methods that may be employed for gene therapy [Table 1].

**Table 1:** Methods for gene therapy

*Gene therapy method*	*Disease under research*	*Research status*
Gene replacement	Leber′s congenital amaurosis	Clinical trial. Interim results obtained
RNA interference	Retinitis pigmentosa	Invitro, animal experiments
	Age-related macular degeneration	Clinical trial
	Retinal neovascularization	Animal experiment
DNA nanoparticle	Retinitis pigmentosa	Animal experiment
*Ex vivo*	Retinitis pigmentosa	Animal experiment

## Gene replacement

In autosomal recessive disorders like *Leber′s congenital amaurosis* (LCA), both copies of the same gene are mutated resulting in defective protein production, similar to a knockout animal model. In gene replacement therapy, a defective gene (protein) causing loss of function is replaced by a normal copy of the gene (protein) delivered to site of action through an attenuated viral vector [Table 1]. Acland *et al* performed successful RPE65 gene replacement therapy in natural canine models of LCA that resulted in restoration of vision, a landmark experiment that prompted clinical scientists to adopt the strategy to restore vision in humans with recessive retinal degenerative diseases.[3]

The first gene therapy clinical trial was started in May 2007 at the Institute of Ophthalmology and Moorfields Eye Hospital, London, UK. Three young adult LCA patients with early onset, severe retinal dystrophy, who had missense mutation in *RPE65* gene were administered single subretinal injection of recombinant adeno-associated virus vector that expressed *RPE65* complementary DNA (cDNA) in one eye, which had worse visual acuity; the contralateral eye was used as a control. Visual function and immune status were assessed after two, four and six months and graded as predefined end points for toxic effects and efficacy for improved visual function.[2] Except for mild self-limiting postoperative intraocular inflammation, all the patients were free of any adverse effects. Although none of the patients showed any change in retinal response to electroretinography (ERG), one of the patients was observed to have significant improvement in visual function and mobility.[2] Similar trials supported by the National Eye Institute were conducted at the Universities of Pennsylvania and Florida.[4,5] After three months of sub-retinal administration of vector-mediated RPE65 gene, patients showed evidence of increased visual sensitivity and activated retinoid cycle however with slow resensitization kinetics of rods, when compared to the control untreated eye.[5] A one-year follow-up has revealed that the patients have not experienced decrease in vision as compared to the short-term follow-up, establishing sustenance of effect of gene therapy.[6] These trials were also independently free from serious adverse effects of vector-based gene delivery thus proving their efficacy and safety.

## RNA interference

While gene replacement is the most suitable method for autosomal recessive diseases where there is no normal protein production, it is not a method of choice for autosomal-dominant (AD) diseases. In AD diseases there is one defective copy that produces mutant protein and one that produces normal protein, resulting in a dominant-negative effect, where the wild type and mutant protein interact with each other resulting in blocking of normal protein function. RNA silencing aims at preventing the mutant gene either from getting transcribed into messenger RNA (mRNA) or preventing mutant mRNA transcript from being translated into a protein [Table 1]. Silencing the RNA is achieved by a number of ways of which small interfering RNA (siRNA) and ribozyme therapy are promising. siRNA are small fragments of RNA that are around 22 nucleotides in length and complimentary to the mRNA of interest. It binds to the cellular proteins forming a complex, where it unwinds and targets the mRNA with the guidance of the complex and completely degrades it. Ribozymes are RNA molecules that act as enzymes. In AD retinal degenerative diseases, ribozymes are directed to neutralize the mutated mRNA transcript thereby inhibiting wild-type mutant protein interaction by delivering of engineered RNA strands or specific binding of the ribozyme RNA to mRNA encoded by the mutated gene or by cleaving target mRNA, preventing it from being translated into a protein. Since mutation heterogeneity is common in autosomal dominant diseases, Millington-Ward *et al*, developed a mutation independent-suppression replacement strategy, which has been evaluated in *in vitro* cell cultures with transcripts derived from rhodopsin and RDS-peripherin genes.[7]

### Other methods

Animal experiments are underway for delivery of DNA using nanoparticles. Recently, nanoparticle-mediated transfer of retinal degeneration slow (Rds) gene in *rds^+/−^* mice, was shown to result in significant structural and biochemical rescue of the disease. ERG showed significant improvement in cone function and fairly good rod response.[8] In 2009, Gregory-Evans *et al* showed in a rat model of retinal degeneration that intravitreal injection of glial cell-derived neorotrophic factor (GDNF) secreting embryonic stem cells could sustain neuroprotective effect on retinal structures for at least three months.[9]

Ocular neovascularization is the central feature in the pathogenesis of ischemic ocular diseases such as *retinopathy of prematurity* (ROP), *proliferative diabetic retinopathy* (PDR) and *age-related macular degeneration* (AMD).[10] While neovascularization occurs in different parts of the eye such as cornea, iris, choroid and retina, retinal and choroidal neovascularization gain pathological importance essentially due to their involvement in retinopathies and AMD, respectively. Vascular endothelial growth factor (VEGF) is a key modulator of angiogenesis and has been shown to be over expressed in ocular diseases in which, angiogenesis is the primary feature. Inhibition of VEGF in a murine model through recombinant adeno-associated virus (rAAV) mediated transfer of gene coding for its receptor, soluble VEGF receptor 1 (sFlt-1) has been shown to reduce retinal neovascularization by 50%.[10,11] Similarly siRNA targeting VEGF has been shown to reduce retinal neovascularization in human cell lines and after adenoviral-induced transgene expression of hVEGF in mouse models. It was also shown to reduce laser-induced choroidal neovascularization, which is often noticed in AMD.[12] An AAV-mediated gene transfer of pigment epithelium-derived factor (PEDF) was shown to reduce choroidal neovascularization significantly in mice exposed to laser for Bruch′s membrane rupture.[13] Pre-clinical and clinical trials using the above-mentioned techniques are underway for other molecules involved in ocular neovascularization, such as protein kinase C (PKC)-β, angiopoitein-1, metalloproteinase-3 etc, in such massive scale that is beyond the scope of this review.

## The future

The recent success of gene replacement therapy for LCA is a big step forward in the field of genomic medicine. These results have enthused the medical community and basic scientists equally and have unveiled the potentials that is in store for the future of medicine. Once these experiments are refined and tailored to the needs of these patients with unambiguous success, nearly 500 eye genetic diseases and 1500 genetic diseases in other parts of the body could be potentially cured.
